# Supplementary material for: Nontrivial Topological Properties and Synthesis of Sn2CoS with L21 Structure
Source: Nanomaterials (Basel). 2023 Apr 17;13(8):1389. doi: 10.3390/nano13081389 (PMC10141049; doi:10.3390/nano13081389)
Supplement: Supplementary file 1 [file nanomaterials-13-01389-s001.zip › nanomaterials-2267985-supplementary.pdf]

Nontrivial Topological Properties and Synthesis of Sn_2CoS with $L2_1$ Structure

Guifeng Chen [1,2,*], Bolin Long [1,2], Lei Jin [1,2], Hui Zhang [1,2], Zishuang Cheng [1,2], Xiaoming Zhang [1,2,*] and Guodong Liu [1,2,*]

[1] Hebei Engineering Laboratory of Photoelectronic Functional Crystals, Hebei University of Technology, Tianjin 300130, China

[2] School of Materials Science and Engineering, Hebei University of Technology, Tianjin 300130, China

* Correspondence: cgfchen@hebut.edu.cn (G.C.); zhangxiaoming87@hebut.edu.cn (X.Z.); gdliu1978@126.com (G.L.)

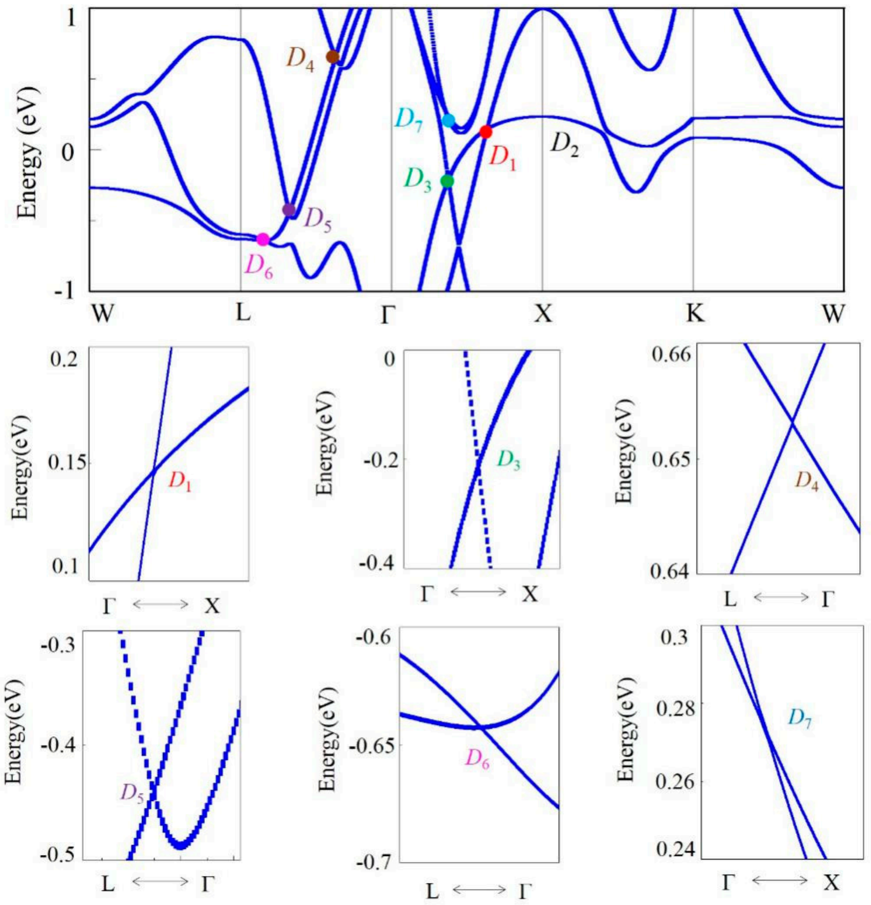

Figure S1 Electronic band structure of Sn_2CoS with SOC. The point and gapped nodal point are indicated by D_1, D_2, D_3, D_4, D_5, D_6 and D_7, and the detail electronic band structure near the above points.

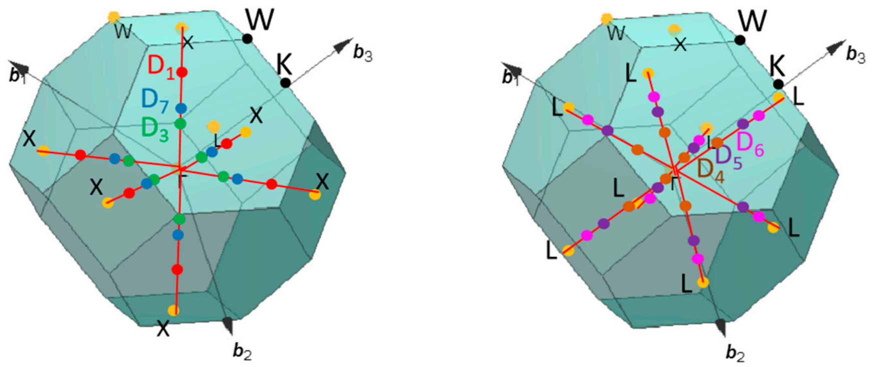

Figure S2 the bulk Brillouin zone of the Sn_2CoS with D_1, D_3, D_4, D_5, D_6 and D_7.

Table S1 The energy level of D_1, D_3, D_4, D_5, D_6 and D_7.

Dirac point	Energy level
D_1	0.146eV
D_3	-0.212eV
D_4	0.653eV
D_5	-0.44eV
D_6	-0.643eV
D_7	0.27eV

Table S2 The magnetic of Sn_2CoS with different DFT+U value.

DFT+U	Magnetic
U0	0
U2	0.59
U4	1.00
U6	1.25
U8	1.64
U10	2.30

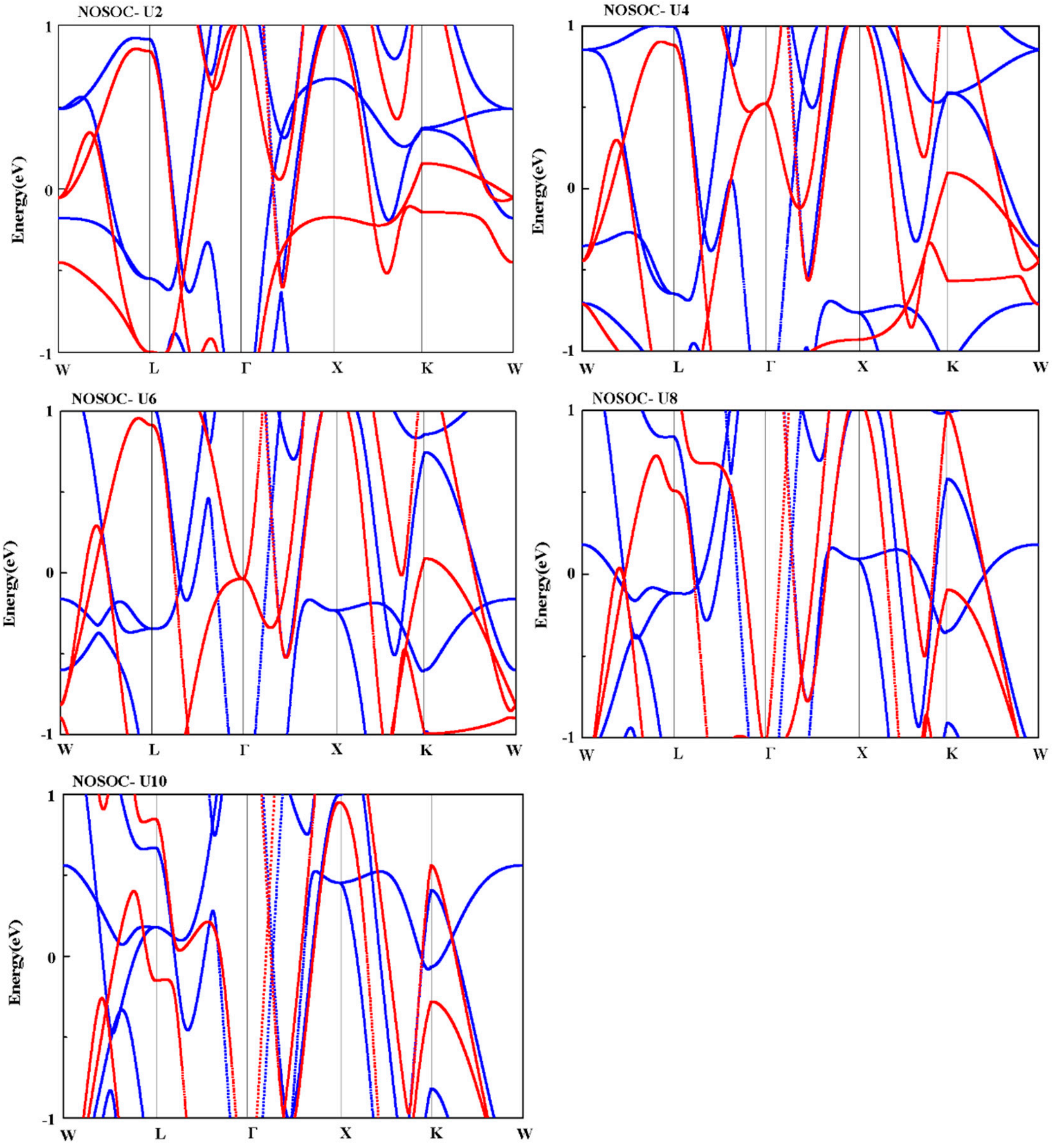

Figure S3 The band structure of Sn_2CoS with different U values.
